# Culture of care: the question of animal agency in laboratory animal science

**DOI:** 10.3389/fvets.2024.1373778

**Published:** 2024-09-10

**Authors:** Katharina Ameli, Stephanie Krämer

**Affiliations:** Interdisciplinary Centre for Animal Welfare Research and 3R (ICAR3R), Justus Liebig University, Gießen, Germany

**Keywords:** human–animal studies, qualitative research, expert interviews, multispecies ethnography, animal ethics

## Abstract

**Background:**

A majority of the current debates in experimental animal science research focus to a large extent on the significance and implementation of the 3Rs principle according to Russell and Burch. In this context, not least due to the EU Directive 2010/63/EU, the concept of a culture of care has become more prevalent. Although animals are essential actors in the field of laboratory science, the discussion around animal agency, as well as the resulting consequences for laboratory animal science, is currently unconsidered.

**Methods:**

The purpose of this qualitative survey was to identify the perception and understanding of professional workers in laboratory animal science regarding the culture of care in general and aspects of animal agency in particular. Using a non-standardized qualitative survey method (topic-oriented, guideline-based expert interviews), persons involved in animal experimentation in different groups (management, science, regulation, and care) were interviewed.

**Results:**

Overall, the results of the qualitative survey showed that animal agency plays a subordinate role in the question of a culture of care in animal research. Although not all groups explicitly applied the construct of animal agency or comparable terminology for this, there were links to the theoretical construct. Overall, the interviews showed a recognized network between humans and animals and that animals can interact dialogically in research. This is justified, for example, by the transfer of emotions from carers or scientists to animals. Nevertheless, a differentiated reflection of an animal’s agency remains disregarded.

**Conclusion:**

The present qualitative survey approached the understanding of a culture of care among experts in the field of animal research. Animal agency does occur in the theoretical reception of the culture of care model. However, it is not conclusively established in everyday practice. Rather, the results lead to the assumption that strategies are being implemented to largely fade out animal agency.

## 1 Introduction

In 2010, the European Union adopted Directive 2010/63/EU, thereby creating an instrument for European member states to implement effective measures to regulate animal experimentation by law. The implementation of the 3Rs concept by establishing a culture of care in the sense of a “Climate of Care” is explicitly mentioned in Recital 31 of the EU directive but has not yet been conclusively established.

A majority of the current debates in animal sciences focus on the importance and implementation of Russell and Burch’s 3Rs principles. The most cited content in the authors’ study relates to the description of the tools for implementing “humanity,” through the 3Rs, which are replace, reduce, and refine. Replace describes a replacement of animal experimentation with an alternative procedure. If this is not possible, the smallest necessity of animals should be used, in the sense of reduce. If experiments must integrate animals, an effort should be made to refine the studies so that the laboratory animals used endure the least possible amount of pain, suffering, or harm. This is in addition to the reduction in stress and the best possible preservation of the wellbeing and welfare of laboratory animals (1).

Welfare “has been used in the animal research literature to mean simply the absence of distress, but it also can be and has been used to refer to a number of different positive mental states-ranging from very mild and brief feelings of comfort; to feelings of great comfort; to satisfaction resulting from eating, drinking, and the fulfillment of certain basic physiologic needs; and to mild pleasures, intense pleasures, feelings of happiness, and happy lives” (2). The attribution of feelings, happiness, and the parameters of wellbeing in laboratory animals is closely linked to the conclusion that “laboratory animals respond to many, if not all, of the activities occurring around them both behaviorally and physiologically” (3).

The importance of human–animal relationships has been shown to have an impact on animal stressors (4, 5). Positive interactions with caregivers reduce abnormal behavior, increase species-specific behavior, and promote coping skills that help attenuate stress reactivity to novel objects or situations (6). Positively oriented interactions with animals also lead to increased morale and job satisfaction among caregivers, resulting in better care and improved animal welfare (7–9). This is linked to current debates on the One Health (10) and One Welfare approach (11).

Russell and Burch’s statements allow for initial conclusions about the complexity of the principles. This implicitly illustrates a necessity for reflection on procedures in organizations and attitudes toward animals and links this to the concept of a culture of care (12).

Culture of care finds its roots in the area of nursing and health promotion with a foundation in communication (13). However, a differentiated view shows more parallels to culturally sensitive nursing. The term is used when people with different cultural contexts interact to build a high-quality relationship in care (14). At its core, it is characterized by trust-building words, gestures, moments, and touches and is linked to an inner attitude. This includes that people, regardless of their worldview, origin, or social position, are valued and that they are treated with empathy and compassion (15). In comparison, the term culture of care is generally used in the laboratory animal community to indicate a commitment to improving animal welfare, scientific quality, care of the staff, and transparency for the stakeholders (Norecopa). The concept describes a transformation of existing routines and procedures toward dialogical processes of negotiation and reconceptualization of all groups involved. All groups involved (management, science, regulation, and care) are understood as multipliers in the 3R implementation and the implementation of a culture of care in the sense of appreciation, care, and the wellbeing of all actors (16). A multiplier is a person or institution through which knowledge and information pass through its dissemination and reproduction to other people and organizations.

In summary, the concept of a culture of care describes (17–19):

- Commitment to the implementation of the 3Rs;
- Creating an appreciative working atmosphere;
- Institutional engagement on behalf of animals (leadership has a key role);
- Motivation building and promotion of creativity of all employees;
- Barrier-free communication within and between all groups of an organization;
- Remodulation of values, beliefs, and attitudes;
- Professional and interactional promotion of all actors;
- Strengthening the self-organization of each individual;
- Lifelong learning in the sense of ongoing training programs for all groups;
- Appreciation of humans and animals.

Russell and Burch’s study contains relevant links to the culture of care, even if it is not explicitly named as such. They aim for the existence of a friendly and constructive attitude toward the animals used, which serves as a means for the (further) development of experimental techniques. This includes the inclusion of philosophies that prohibit attributing consciousness to animals (12). One approach to countering this is Singer’s animal ethics, which fundamentally assumes that the sentience of animals is linked to consciousness (20). When applied to research with animals, this means—despite the difficulty of objectification—that a debate on the question of animal consciousness and its implications for scientific research is necessary to think about the inclusion of this aspect within experimentation.

The 3Rs are used all over the world, although it must be emphasized that considering the 3Rs alone can lead to a rationalization of the concept without embedding it in its original context. The quotations usually refer to Refinement, Reduction, and Replacement. However, Russell and Burch’s ideas on humanity and inhumanity with reference to subjectively perceived but ignored parameters remain unknown. Consequently, the different reception of the authors can lead to the fact that the highest goal of Russell and Burch, namely the complete renunciation of sentient beings, cannot be achieved (12). With reference to this, Russell and Burch also refer to a fundamental objective of their work as being “to create a new discipline of applied science.” The aim of the authors is to counter direct contingent cruelty (inhumanity) toward animals in biomedical research by integrating reflection processes from an animal’s perspective (21). These can be understood as relevant characteristics of a future culture of care. In conclusion, it is therefore logical that the perception of these research findings has criticized problematic situations due to the treatment of laboratory animals (22, 23). These have contributed to further reflection on established (social) organizational culture(s) of animal research and a discussion and (self-)reflection about the culture in animal research (17).

There have been an increasing number of conferences and workshops about the culture of care. Nevertheless, the current debates linked to the 3Rs and culture of care exclude an essential part, namely, the deeper and differentiated analysis of animal agency (16).

This exclusion reveals a major shortcoming of the conceptualization. The microperspective view of animals does not go beyond a mere discussion of the broad term “well-being” or the rationalized aspects of the 3R principles. The idea of animal agency in animal sciences is the ability to make decisions based on the animals’ interests and respect animal rights (24, 25). Legal animal rights linked to animal agencies ensure that the interests and personalities of animals matter and that this is qualitatively balanced and discovered in research. Moral status can therefore be equated with the moral consideration of interests (25). Although Russell and Burch and the concept of culture of care do not explicitly name the construct of animal agency, their explanations do already show links to its concept. For example, Russell and Burch explicitly address the network of effects and interactions that arise between humans and laboratory animals (1). Their first thoughts can be linked to current debates about animal agency within the field of human-animal studies, e.g., the social sciences, humanities, or interdisciplinary human–animal studies. Russell and Burch recommend an integration of the ambivalence between humanity and inhumanity within research. The characteristics of a culture of care address an appreciation of animals, an engagement with them, and a remodulation of beliefs, values, and attitudes.

Animal agency as a theory is described as a construct that integrates free will, ability, rationality, mind, morality, and subjectivity for animals (26). The first debates focused on agency were human-centered and were oriented toward anthropological approaches and questions about social and political organization. The agency is species-bound and contextualized through dichotomies, power relations, and moral concepts at the interface of the human–animal bond (26–28). Following on from philosophical and sociological approaches, animal agency can be defined as acting, action, and influence with the inclusion of animal morality (26, 29–32). It integrates an acceptance of sense and intuition and the various emotions and faculties, such as love, memory, attention, curiosity, imitation, and reason (29).

Russell and Burch also address the consideration of animals’ emotions as an integral part of animal research. Animals show physical and psychological species-specific and individual behavior and expression, which is linked to ethical behavior. McFarland and Hediger describe the gorilla Binti as an example of this. Binti took a boy who had fallen into her enclosure in her arms until the animal keepers took the child. Binti acted like a moral being, which allowed the authors to see evidence of the presence of animal agency (26, 33–35).

Linguistic research shows that animals participate in communicative and symbolically based worlds in various and very fundamental ways (36). Ethological studies confirm the species-specific and individual behavioral and expressive patterns of animals (27).

Animal agency is formed within social interactions, in which animals can act socially and enter into relationships. Interrelated behavior is linked to the existence of a social relationship and social action through feelings, moods, body positions, body language, and facial expressions (37–40). Animal agency becomes apparent through four elements: agency through time and space, practice and routine, agency in the social environment, and agency through social norms. Recognition of this is linked to empathy for different species (41). This allows for a discussion on what it is like to be (like) the other. However, it does not answer the question of “what it is to be ‘with’ the other” (42). Multispecies ethnography is also linked to this approach, which is a research method that seeks to combine animal perspectives with interdisciplinary-oriented research approaches by integrating (auto-)ethnographic analyses of human and laboratory animal interaction(s) (43).

In relation to the theoretical reception of animal agency in the literature, it can be characterized and summarized as the capacity of animals to make decisions, determine and take action, and organize themselves individually and as groups. It includes the recognition of animals’ voices (44) and their capacity to act intelligently, rationally, and intentionally (45).

In this context, the analysis of stress and behavioral aspects such as fear or other mental states of animals has not been sufficiently revealed in animal research (2). Nevertheless, it is therefore very impressive that animal perception and personality are named and linked to philosophies that attribute consciousness to animals. Consciousness means that the interests of all sentient beings are morally significant. Sentience describes the ability to have positive (pleasant and attractive) and/or negative (unpleasant and aversive) experiences, both physical and emotional. They arise in interactions and through reactions with the environment. Indicators could be signs of rejection by withdrawing, refusing, or screaming, which shows an unwilling participation (46). Therefore, a deeper analysis and understanding of what that means are missing. Researchers should look “for what is being unconsciously ignored” (1). With reference to the theoretical concepts of animal agency and the 3R concept, an animal’s perspective is not given sufficient consideration. Attempts are indeed being made to strengthen animal welfare and well-being through, for example, ethical application formats and the assessment of stress. However, a critical view of this addresses an ignorance of parameters that go beyond animal welfare parameters (47). These include, for example, perceived personality traits of animals or exhibited behaviors that are not relevant to the planned research project. As a consequence, animals in the animal sciences are not conclusively understood as physical-spiritual entities that can act meaningfully and exert a reflexive and active influence on their environment through individual self-determination (44, 45).

Although Russell and Burch’s study and the concept of culture of care can be linked to the ideas of animal agency, the interdependence has not been analyzed, nor has the EU directive or the concept of a culture of care integrated animal agency sufficiently.

At present, there are no empirical data about animal agency in the context of a culture of care in Germany. This means that we currently do not know what animal agency means for people or how or whether animal agency is taken into account in practice.

With the help of explorative qualitative research (expert interviews in various groups: management, scientific, regulation, and care), an animal agency within the concept of culture of care was examined (19). This article examines the specific research question of animal agency from the perspective of employees at different levels at institutions involved in animal research and offers the first hypothesis about the implementation of this theoretical approach in practice.

This article addresses this research gap at the interface of the 3Rs, culture of care, and animal agency.

## 2 Materials and methods

### 2.1 Objective

Data collected are based on a qualitative approach with non-standardized, topic-oriented, guided expert interviews. The interviews were conducted with people associated with animal experiments at various levels (management level, scientific level, supervisory level, and care level). This qualitative approach allows for theoretical, methodological, and methodical approaches to social reality (48, 49) by analyzing the perception and understanding of culture of care in general with regard to animal agency in particular.

The research questions are intended to highlight the characteristics of a culture of care from a personal and institutional perspective. Sub-questions differentiate the research and offer insights into thoughts and beliefs about animal agency.

The studies involving human participants were approved by the Justus Liebig University Giessen. The study involving human participants was conducted in accordance with the European General Data Protection Regulations and with the Code of Ethics of the German Sociological Association.

### 2.2 Methods

Since the field of research on animal agency is currently very lacking in Germany, an explorative approach was chosen. Explorative expert interviews are particularly suitable when there are few or no examples of theoretical or empirical data available. All actors involved offered multi-layered insights and perspectives that included knowledge, action, and their social meaning (48).

The qualitative approach with small cases does not aim to generate countable or measurable results. Rather, individual expert perspectives and experiences are considered within the contexts, conditions, strategies, and consequences. These offer insights into a perception of animal agency in animal sciences (50). The sample is based on theoretical sampling. Participants were selected based on their potential for developing and refining theoretical concepts. Experts with a perception of the theoretical framework were chosen, as well as experts with no perception. This assumes that the sample size was achieved because additional data will not produce any relevant new findings (51).

### 2.3 Field excess

All participants were unknown to the interviewer. The participants were recruited via stakeholders and distributors and could decide whether they wanted to participate or not. All participants received written information about the procedure and signed a consent form regarding their participation.

Field access was very challenging, especially for the group of scientists as well as the management group. More people had to be approached, as there was less willingness to participate. In some cases, scientists were instructed by their managers not to discuss any information about individual research projects. The regulatory group and the care group showed great openness and commitment to participation.

### 2.4 Sampling

Between October 2020 and January 2021, a total of 14 experts in animal-based research were interviewed. They came from multiple labs and institutions throughout Germany. Experts worked with laboratory animals and were employees at institutions that are actively involved in animal research. These were assigned to four organizational groups: management, science, regulatory, and care. The management group included all experts in a management function, such as a working group leader, veterinary manager, or head of an animal facility. The science group included all experts with a scientific focus, such as postdocs and doctoral students or technicians. The regulatory group included all experts who carry out supervisory activities in the context of animal research, such as animal welfare officers or authority representatives. The care group included all experts who are responsible for the care of the animals, such as animal caretakers. It should be noted that the roles of experts may overlap between the groups.

Methodologically, the sample was chosen in such a way that “every reality of the phenomenon under investigation” was present (52). Three people each were interviewed for the management and scientific groups, and four people each were interviewed for the care and regulatory groups.

In summary, the interviews addressed the individual process of working with laboratory animals over time, the definition of culture of care, and links to animal agencies.

All interviews conducted were transcribed and provided with field notes and memos. Care was taken to consistently anonymize all participants. The participants needed to suffer no disadvantages from participating in the research, which is why protection was assured in writing. All interviews were productive and based on trust (53).

### 2.5 Evaluation

The analysis of the culture of care required a complex consideration of all groups. The basis of the analysis was the assumption of a relationship between humans and animals (54).

Analysis with grounded theory using MAXQDA, a qualitative data analysis software, allowed for data collection with expert interviews and by theoretical sampling—simultaneously coded and analyzed. This process was guided by theory and allowed for the integration of animal agency from the experts’ perspective (55). All codes were generated when aspects of animal agency were addressed within the interviews. The interview passages of the participants within the text were translated from German to English.

## 3 Results

This section is divided into different groups. It provides a concise and precise description of the results and their interpretation, as well as the conclusions. The results of the qualitative interviews show a differentiated representation of animal agency about a culture of care. For a better understanding of the data, the presentation of the results was first divided into individual groups. This was followed by an overarching conclusion for all groups to classify the significance of agency from a meta-perspective.

### 3.1 Management group

The management group emphasized less implementation of the concept of culture of care. Two managers were barely aware of the concept before the interview. One manager emphasized that the culture of care is already being integrated into his understanding and everyday work. Communication plays a key role in the care of the animals, as well as the legal framework and financial planning of the activities within the organization. In the argumentation for implementing a culture of care, systemic parameters were highlighted as barriers, for example, the pressure to publish or inconsistent jobs. Job practice in the German science system causes negative effects on humans and animals, as the following quote illustrates:

“The most glaring effect is that research results are generated and animals are used for them that actually have no value because they are based on a false premise. Then the animals are virtually useless, in the best case they have died, in the worst case they have been subjected to some painful or stressful procedure. Something did come out of it – in the sense that something was published in the end – but it did not actually contribute anything to the actual gain in knowledge.” (Animal House Direction).

The management group also highlighted the necessity of education to implement the 3Rs and a culture of care. It is fundamental for animal welfare and stimulation of internal reflection and individual roles. These also refer to a fundamental questioning of one’s activities. In this context, the question of the animal as a product was also raised in the sense that animals can be used strategically and be scientifically useful for scientific output.

A quote from one manager illustrates this:

“I think everyone who does animal experiments sooner or later comes into a crisis. I have experienced these crises several times in my life and actually not a month goes by where I do not ask myself: Is this actually justifiable?” (Professor).

In this context, the person points out that this reflection includes the fact that the individuality of the animals exists:

“The individuality of the laboratory animals, which we have tried to eliminate for decades, is still there.” (Professor).

Although shades of animal agency became visible, the term itself was not mentioned. Therefore, animal perspectives were not given deeper consideration.

### 3.2 Regulatory group

The regulatory group emphasized the importance of close contact with other organizational groups involved, especially with other regulatory actors, scientists, and management. The differentiated analysis indicated self-discipline and the ability to reflect as markers for a culture of care, as well as respect for animals and communication between all groups.

An animal welfare officer stated the following in connection to animal agencies:

“An essential aspect of implementing a Culture of Care is a change of perspective. I believe that in order to really process things.” (Animal Welfare Officer).

Giving animals a higher status and reflecting their perspective within experimental procedures helps to improve methods. Furthermore, a reflection on subjectivity within research gives new insights into interpretations from a human-centered perspective.

One expert of the regulatory group emphasized the fundamental question of indispensability and the ethical question of the “self-evident” use of animals (agency representatives). Shades of the animal agency were recognizable. However, animal agency in a deeper sense was seen critically by an authority representative:

“But to what extent animals are able to influence something like that, I do not know, so with rats, rats are intelligent anyway, I do not know if rats are willing to influence anything. Monkeys maybe.” (Authority Representative).

### 3.3 Science group

Culture of care was largely associated in the science group with providing animal welfare. In this context, communication between all groups was described as relevant to implementing animal welfare. Animal caretakers were named as mainly being responsible for ensuring animal welfare. References to animal agencies were not directly made. However, the effects of interactions between humans and animals were emphasized and reflected upon.

“When I deal with the animal in a calm and balanced way, radiating a certain aura. Then the laboratory animal will also respond to that. But if I’m totally stressed because I want to be ready quickly, because I have to go to the lab somehow after the animal house, then that also has a very strong effect. That’s why the most important thing is to always remember that you are really working with living beings and that you should take your time and rest for it. Even if that is sometimes difficult in a workday.” (PhD Student).

Reflection on working with animals played an important role in the science group. Emotions of humans and animals were named as being interwoven. This emotional bond becomes more relevant when animals are killed.

“I’ve also had everyone tell me that there is actually no one who is completely unaffected. You just learn to deal with it, to put it that way.” (PhD Student).

Both quotations give insights into the emotions of workers and their connection to animals. By addressing this, an inner conflict can be seen. Nevertheless, the quotes spoke to a strategy to deal with these emotions: focusing on objective parameters within research and accepting the facts.

Any subjective parameters that may arise were not considered. It is therefore not surprising that animal agency is seen as a form of influence but would not actively take place:

“I do believe that animals can actually influence a great deal, but of course they do not actively do so.” (PhD Student).

The science group showed shades of recognition of animal agency but pushed aside individual needs and emotions, which reflected on the animals’ needs, emotions, or personalities.

### 3.4 Care group

The care group showed a differentiated view and mostly supported the implementation of the 3Rs. The majority of the participants did not know the concept of the culture of care. Furthermore, the construct of agency was not explicitly mentioned. Nevertheless, the care group saw themselves as having a high level of responsibility for the animals:

“We have a certain responsibility as animal keepers and above all we really provide some protection for the animal during our work, educate ourselves to then be able to handle the animal better.” (Animal Keeper).

This also revealed an inner conflict in the form of an ambivalence dilemma, which was also named earlier in the science group. The strategy for dealing with the activity and the killing of the animals places higher stress on the care group. This requires finding strategies to deal with this:

“When I really get out of my area, I can switch it off and then I do not think about the part I did there at work. […] It developed relatively early in the training that you have to realize that. I have a different opinion in my head about the subject than how I actually work. That developed relatively early in my life: This is work and this is private life.” (Animal Keeper).

As was already mentioned in the science group, the ambivalence dilemma was overlooked or even ignored. The attributions and social constructions of the participants to the animals showed links to the construct of animal agency, always in connection with interactions:

“Mice also show sympathy. If someone is hectic, the mice are hectic and you notice it when an animal keeper changes. If one animal keeper is on holiday and someone else takes his place, the mice are also a bit more hectic when they are first moved.” (Animal Keeper).

It can be concluded that animal agency is linked to complex emotional conflicts, which are also described in the scientific community as compassion fatigue syndrome (47, 56). The distance or absence of emotions is described as an element in being able to perform certain activities, such as euthanizing animals. The exclusion of individual emotions logically prevents the ability to empathize with other beings. It is therefore not surprising that animal agency has not been noted more deeply, as the following quote states:

“I have to be honest and say that I have not yet noticed this in mice.” (Animal Keeper).

## 4 Discussion

Overall, the results of the qualitative research showed that animal agency plays a subordinate role in the question of a culture of care in animal sciences. The lack of consideration of animal agency is consistent with the literature in animal sciences and highlights a blind spot in the current debates about culture of care within the scientific community of animal research.

Although no interviewed groups explicitly applied a recognition of animal agency in everyday practice, relevant links to its theoretical construct can be made.

Overall, the results showed a general recognition of the networks between humans and animals and that animals are attributed to an agency in which they interact dialogically. This is justified, for example, by the transfer of emotions between care workers, scientists, and animals.

Named external and internal conflicts regarding the activity and killing of animals allow for further conclusions about the concept of animal agency in animal sciences. The participants explicitly mentioned strategies of demarcation to counter discrepancies with their own values. It can be assumed that this is one reason why the agency occupies less space within the concept of culture of care.

The results reveal a major issue regarding values and beliefs in connection with professional identity (47).

However, objectified research is emphasized as an essential quality criterion and strategy, so that essential aspects, such as emotions or individual values, are automatically excluded—as Russell and Burch already noted. In recent debates, scholars have pointed out potentially subjective and ignored parameters (25). For example, Helena Pedersen named the fragility of objectivity in an exemplary research study with chickens. Although the chickens involved behaved in an experimental situation, they were able to influence the experiment through their agency. The researcher’s subjective assessments became visible in her reflection but were not made available to the scientific community (57). Another example is that the gender of workers had an influence on the behavioral parameters of tested animals (58).

Although aspects of the animal agency have already been discussed, no research about the animal agency or subjective parameters in animal sciences has been adequately linked beyond usual standards.

Thus, if we assume that consideration of animal agency is practiced on an individual basis, this aspect is supported by our research.

## 5 Conclusion

Overall, both the theoretical reception of animal agency and the results of the qualitative data offer insights about a missing perception of animal agency in debates around the culture of care. As a result, culture of care could, in its current form, result in the establishment of procedural rules through the use of animals in animal research.

However, a deeper and more reflective approach offers animal agency as one core element of a culture of care. Even though it is not referred to as an animal agency within the concept, animals and their appreciation are included. The animal agency goes beyond the legal regulations of institutions, general acceptance of the 3Rs, and ensuring animal welfare. It means that a transformative attitude on the part of individuals and institutions is necessary if animal agency is to be taken into account.

However, the study aimed for an understanding of the characteristics of a culture of care among professionals. Nevertheless, an underrepresentation of animal agencies in animal research was noted, and a consensus about the absence of a deeper influence on animals was highlighted.

With reference to the theoretical reception of Russell and Burch and the concept of culture of care, an increasing lack of consideration of animal agency remains, which leads to a blind spot in attitudes toward animals. For animals, this means that their character, ability to interact, individuality, needs, and voice are not sufficiently holistically heard within animal research. To address this gap, a transformation and cross-disciplinary work may be beneficial. Using multispecies ethnography allows for a new possibility of approaching animals. Multispecies ethnography describes an ethnographic method that observes and reflects human–animal bonds. By conceptualizing animals as social actors with agency, this helps question established routines and methods. It is generally recruited for research that acknowledges the interconnectedness and inseparability of humans and other life forms of the more-than-human world, such as animals (59). Multispecies ethnography offers to reconstruct interactions and social relationships between humans and animals in the animal sciences. (60)

In the first step, the methodology allows for a shift by including people writing about people and animals. This requires that common and established rules are broken and previous knowledge is questioned to make it useful for analyses of animal agency. The heuristic tool offers seeing everything through the eyes of an animal. It combines sensorial, visual, and video-based research methods with an ethnographic focus and consideration of different species. Animals are perceived as living beings with their own experiences, sensations, perspectives, and interests (61, 62). Consequently, the subjectively perceived parameters of researchers are highlighted by integrating the emotions, feelings, and perceptions of people working with animals in the animal sciences (56). Regarding and respecting animal agency in labs will allow animal sciences to re-think and reflect upon organizational structures and open their expertise to animals’ perspectives. Integrating this allows for a transformation in organizational development for humans and animals. It is, therefore, necessary for further research projects to address concrete markers of how a consideration of animal agency can be integrated within laboratory animal research.

To address this gap, reflection questions are required to implement a critical and reflective perspective on the animal agency.

1. What is the use of animals helpful for?
2. How are interactions between humans and animals in laboratory animal research organized?
  - What is the value of observing animal interactions, and how are they categorized?
  - What interactions do animals show with humans, and how are these interpreted by the caregivers, always in reference to their values, emotions, and beliefs?
3. What emotions do interactions with animals release in the caregivers?
  - Which emotions are recognized in the perceived behavior of joy in animals?
  - Which emotions are recognized by the perceived behavior of fear or stress in animals?
  - Which emotions are recognized when animals are killed (after they have been cared for)?

4. Which forms of consideration of the interest and personality of animals are considered in everyday practice?

- What consequences does this consideration have for the regular procedure?
- How are the interests and personalities of animals considered in relation to aspects that go beyond animal welfare?
- How are animal-perceived “voices” considered within concrete animal experiments?

## Data Availability

The datasets presented in this article are not readily available because the participants did not allow to share personal data to other researchers. Requests to access the datasets should be directed to KA, katharina.ameli@vetmed.uni-giessen.de.
